# Cryo-EM structures of intact V-ATPase from bovine brain

**DOI:** 10.1038/s41467-020-17762-9

**Published:** 2020-08-06

**Authors:** Rong Wang, Tao Long, Abdirahman Hassan, Jin Wang, Yingyuan Sun, Xiao-Song Xie, Xiaochun Li

**Affiliations:** 1 Department of Molecular Genetics, University of Texas Southwestern Medical Center, Dallas, TX 75390 USA; 2 Eugene McDermott Center for Human Growth and Development, University of Texas Southwestern Medical Center, Dallas, TX 75390 USA; 3 Department of Biophysics, University of Texas Southwestern Medical Center, Dallas, TX 75390 USA

**Keywords:** Cryoelectron microscopy, Membrane structure and assembly, Permeation and transport

## Abstract

The vacuolar-type H^+^-ATPases (V-ATPase) hydrolyze ATP to pump protons across the plasma or intracellular membrane, secreting acids to the lumen or acidifying intracellular compartments. It has been implicated in tumor metastasis, renal tubular acidosis, and osteoporosis. Here, we report two cryo-EM structures of the intact V-ATPase from bovine brain with all the subunits including the subunit H, which is essential for ATPase activity. Two type-I transmembrane proteins, Ac45 and (pro)renin receptor, along with subunit *c”,* constitute the core of the c-ring. Three different conformations of A/B heterodimers suggest a mechanism for ATP hydrolysis that triggers a rotation of subunits DF, inducing spinning of subunit *d* with respect to the entire c-ring. Moreover, many lipid molecules have been observed in the Vo domain to mediate the interactions between subunit *c*, *c”*, (pro)renin receptor, and Ac45. These two structures reveal unique features of mammalian V-ATPase and suggest a mechanism of V1-Vo torque transmission.

## Abstract (web summary)

The vacuolar-type H^+^ -ATPases (V-ATPase) hydrolyze ATP to pump protons across the plasma or intracellular membrane. Here authors report two cryo-EM structures of the intact V-ATPase from bovine brain with all the subunits including the subunit H, which is essential for ATPase activity.

## Introduction

The pH homeostasis is essential for the physiological function of cells and cellular organelles. An acidic pH is important for many physiological processes, such as renal acidification, bone resorption, and sperm maturation. V-ATPases localize to the plasma membrane, endosomes, and membrane vesicles and play an important role in proton homeostasis^1–4^. V-ATPase is involved in autophagy^5^, metabolism^6,7^, and neurotransmitter secretion^3^. Recycling of cell receptors is facilitated by V-ATPase through endocytosis^8,9^. For example, the low pH generated by V-ATPase promotes the dissociation of ligands, such as mannose 6-phosphate receptor^10^, which recognizes lysosomal enzymes in Golgi and releases them in prelysosomes by its multiple repeats, facilitating the receptor recycling^11^. Moreover, V-ATPase activity is involved in Wnt, mTOR, and Notch signaling^12–14^. The aberrant activity of V-ATPases in humans is associated with many pathological processes and diseases, such as tumor metastasis, cutis laxa, distal renal tubular acidosis, autosomal recessive osteopetrosis, infectious diseases, and neurodegenerative diseases^3^.

Mammalian V-ATPase is a rotary machine made up of two domains: the ATP-hydrolytic V1 domain and the proton-translocation Vo domain. The V1 domain consists of three catalytic AB heterodimers that form a heterohexamer with threefold rotational pseudosymmetry, three peripheral stalks each consisting of the subunits EG, one central rotor including subunits D and F, and the regulatory subunits C and H; the proton-translocation domain Vo consists of the proton transport subunit *a*, a ring of proteolipid subunits *c*_9_*c”*, rotary subunit *d*, and subunits *e* and Ac45. The subunits B, C, E, G, H, *a*, *d*, and *e* of mammalian V-ATPase have multiple isoforms in various tissues and cells^15^ causing a technical barrier to study its structural and biochemical features due to heterogeneity.

The V-ATPase is structurally and evolutionarily related to the F_1_F_0_-ATP synthases and the archaebacterial ATP synthases termed V/A-ATPases^2^. The composition of the mammalian V1 domain is similar to that of the yeast domain, which is a well-characterized model for V-ATPase studies. The c-ring of yeast’s Vo domain contains eight subunit *c*, one subunit *c’*, and one subunit *c”*. In the V/A-ATPase, there are two subunit E and two subunit G in the V1 domain, and twelve subunits *c* that form a homogeneous c-ring in the Vo domain. Structural insights into V-ATPase function has been gleaned from structures of the intact V-ATPase from *Saccharomyces cerevisiae* (scV-ATPase) at 7-Å resolution^16^, structures of the intact V/A-ATPase from *Thermus thermophilus* (ttV/A-ATPase)^17^, cryo-EM structures of the scVo domain at atomic resolution^18,19^, and most recently published, the structure of rat V-ATPase with its bacterial inhibitor SidK^20^. However, the intact structure of a mammalian V-ATPase with the ATPase activity is still unknown.

In this paper, we purified the intact V-ATPase from bovine brain with the specific ATP hydrolysis activity at about 1.4 μmols of Pi·mg of protein^−1^·min^−1^ and report two cryo-EM structures in the distinct rotational states at overall 3.4-Å and 3.8-Å resolution, respectively. The structures show two unique mammalian V-ATPase components, Ac45 and (pro)renin receptor (PRR) in the Vo domain. This structural work reveals a high-resolution map of the V-type ATPase of bovine brain showing the interaction details between PRR and Ac45. It will also aid in the design of small molecules for the treatment of related human diseases.

## Results

### Overall structure of the *Bos taurus* V-ATPase

**Fig. 1:**
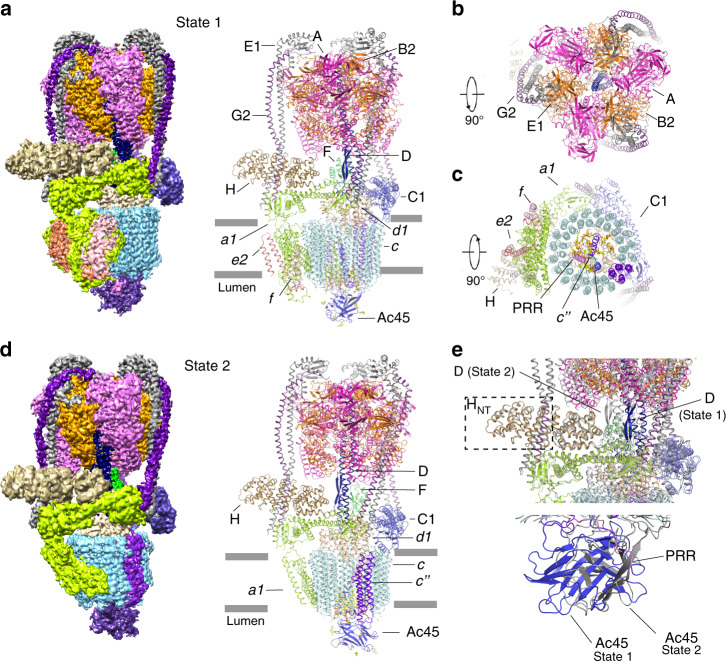
Overall structure of V-ATPase from bovine brain. **a** Overall cryo-EM map and structure showing V-ATPase in state 1 viewed from the side of the membrane. **b** The cytosolic view of the V-ATPase. **c** The luminal view of the V-ATPase. **d** Overall cryo-EM map and structure showing V-ATPase in state 2. Each subunit is colored and indicated. **e** The structural comparison of the two states.

The *Bos taurus* V-ATPase (btV-ATPase) was purified from bovine brain as previously reported^21^. The resulting complex was further purified by gel filtration in the presence of 0.1% CHAPS and 0.004% glyco-diosgenin (GDN) (Supplementary Fig. 1a). The purified protein exhibits high ATPase activity and can be inhibited by bafilomycin A1 in vitro (Supplementary Fig. 1b). The identity of each component of the btV-ATPase was confirmed by mass spectrometry following SDS-PAGE (Supplementary Table 1). This complex was prepared on grids and subject to cryo-EM. The btV-ATPase particles are homogenous showing clear features in the cryo-EM images, making them suitable for high-resolution structure determination (Supplementary Fig. 2). The 3D classification enabled us to distinguish two different states. The 3D refinement of individual classes yielded a resolution of 3.4 Å (state 1) and 3.8 Å (state 2) (Fig. 1a–d). The homology models for subunits A-H of V1 and subunits *a, c, c”, d, e* of Vo were generated by MODELLER^22^ based on the multiple sequence alignments between the bovine subunits and the corresponding subunits of previously determined structures (Supplementary Fig. 3).

The local resolution of the V1 domain is better than that of the Vo domain in both states 1 and 2 (Supplementary Fig. 4). To improve the quality of the Vo and V1 density, we performed a local refinement by specific Vo and V1 masks, respectively, which resulted in a density that shows clear features of the side chains, helping the unambiguous assignment of most residues (Supplementary Figs. 5 and 6). We built B2, C1, E1, G2, *a1, d1*, and *e2* into the final model based on mass spectrometry results and the expression distribution in brain tissues^3^. The maps after local refinement were merged by “phenix.combine_focused _maps” for model building (Supplementary Fig. 6 and Supplementary Table 2). The overall dimensions of btV-ATPase are 160 Å*100 Å*270 Å. Due to the higher resolution of state 1, we discuss the structure of state 1 in the following section, except for the structural comparison of state 1 and state 2.

The subunit H is crucial for the V-ATPase activity^23,24^. A structural study showed that without the Vo domain, subunit H can bind to the A_3_B_3_ hexamer to prevent the rotation of V1 domain, thereby blocking ATPase activity^25^. In both states of our study, subunit H is well determined in the cryo-EM map (Fig. 1a, d). Structural comparison did not reveal notable conformational changes in subunit H with a root-mean-square deviation (RMSD) of 0.656 Å for 365 Cα atoms. The N-terminal helices of subunit H (H_NT_) bind to the subunits E1 and G2 (dash box in Fig. 1e) and—along with the C-terminal helices of subunit H (H_CT_)—interact with the cytosolic domain of subunit *a1* (Fig. 1e). This contact may keep the subunit *a1* in a certain conformation conducive for proton translocation.

### A_3_B_3_ hexamer for ATP hydrolysis

**Fig. 2:**
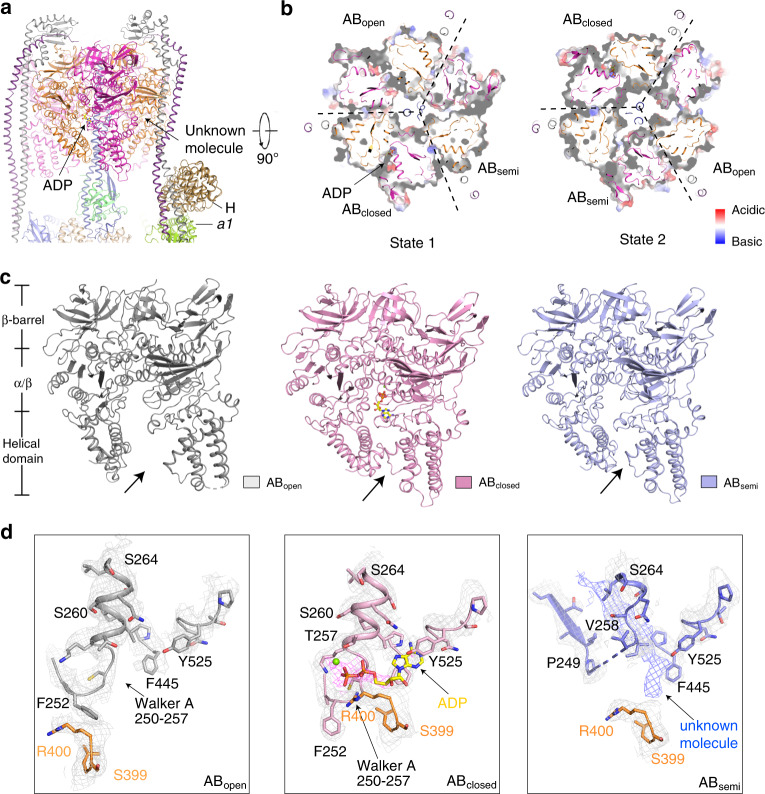
Structure of the V1 domain showing three AB heterodimers with distinct conformations. **a** Overall structure showing the V1 domain. **b** The cytosolic view of the V1 domain in state 1 (left) and state 2 (right). **c**, **d** The structural comparison of AB_open_, AB_closed_, and AB_semi_ in state 1. The domains of the AB heterodimer have been indicated in the left side of panel **c**. Cryo-EM maps in state 1 are shown at the 4σ level.

The A_3_B_3_ hexamer hydrolyzes ATP to provide energy for the rotation of the Vo domain (Fig. 2a, b). Both the A and B subunits have three domains: an N-terminal β-barrel domain, a middle nucleotide-binding α/β domain, and a C-terminal α-helical domain (Fig. 2c). ATP hydrolysis occurs at the interface between the nucleotide-binding α/β domains of subunits A and B^26^. In state 1, three AB heterodimers exhibit distinct conformations (AB_semi_, AB_closed_, and AB_open_) (Fig. 2c; Supplementary Fig. 7). The structural comparison of the AB heterodimers with those of ttV/A-ATPase shows that the three AB heterodimers share a similar conformation with AB_semi_, AB_closed (ADP-bound)_, and AB_open_ of ttV/A-ATPase with RMSD at 2.0 Å, 0.8 Å, and 0.9 Å, respectively (Supplementary Fig. 8). AB_open_ has a pocket open to the cytosol showing a high affinity to accommodate an ATP molecule^26^. Density representing the di-phosphate of ADP with magnesium was found in AB_closed_ (Fig. 2d) that binds ADP in ttV/A-ATPase^17^. Therefore, we built an ADP with a magnesium ion into AB_closed_ (Fig. 2c, d).

Notably, an unknown density was observed in AB_semi_ (Fig. 2d). This unknown molecule binds to a hydrophobic area of subunit A of AB_semi_, and the Walker A motif (G^250^AFGCGKT^257^) of this subunit in the cryo-EM map is not clear (Fig. 2d). The residues 258–264 in the AB_semi_ form a loop to accommodate this unknown molecule; by contrast, these residues form an α-helix in AB_closed_ and AB_open_ (Fig. 2d). It is possible that it represents a nucleotide, since the AB_semi_ exhibits an intermediate conformation for releasing ADP. This small molecule also may be a CHAPS detergent or another unknown molecule. Further investigation is required for identification of this molecule. The C-terminal α-helical domains of AB_semi_ and AB_closed_ are shifted by ~10 Å compared with those in AB_open_ (Fig. 2c), demonstrating substantial conformational plasticity in the A_3_B_3_ hexamer. The structural analysis shows that the AB_semi_, AB_closed_, and AB_open_ heterodimers share similar conformations in states 1 and 2 (Supplementary Fig. 7).

### Overall structure of Vo domain

**Fig. 3:**
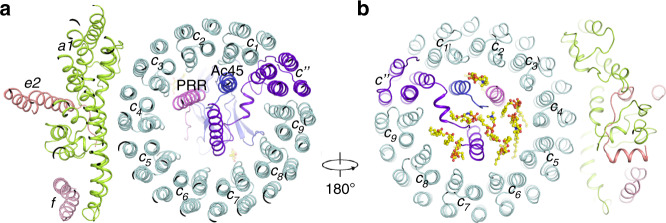
Structure of the Vo domain with its associated lipids. **a** Overall structure showing the Vo domain. **b** The luminal view of the Vo domain in state 1 showing the lipids that are involved in the Vo assembly. The putative PCs are shown in yellow sticks.

**Fig. 4:**
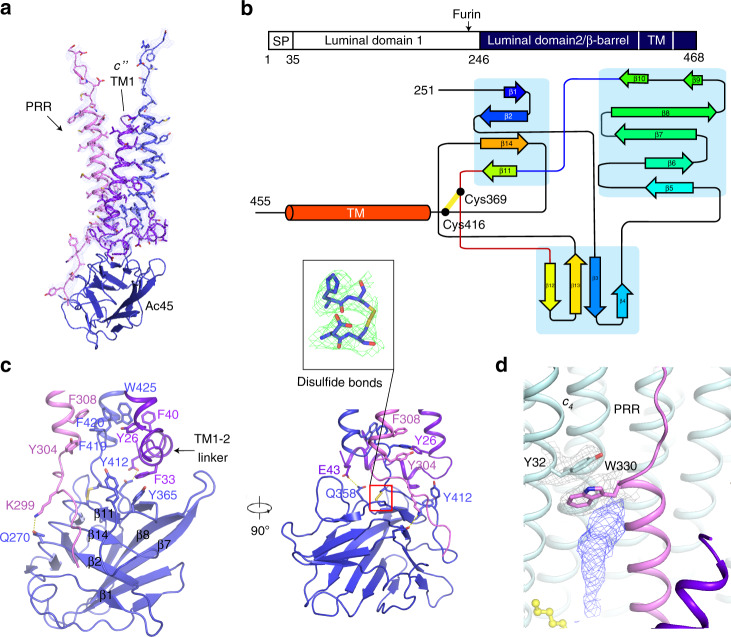
Structure of the core of Vo domain. **a** The core of c-ring. Cryo-EM map of TMs of PRR, Ac45, and c” in state 1 is shown at the 5σ level. **b** The topology diagram of Ac45. The schematic diagram of full-length Ac45 is shown on the top. **c** The interaction details of luminal peptide of PRR with Ac45 and TM1-2 linker of *c*”. The individual β-strand of Ac45 is labeled. The cryo-EM map of the disulfide bonds is shown at the 5σ level. **d** The interaction details of unknown lipid-mediated PRR and *c*_4_ interaction. Cryo-EM maps are shown at the 5σ level.

The subunit composition of the Vo domain considerably differs among species. The Vo domain of ttV/A-ATPase includes subunits *adc*_12_, whereas the yeast V-ATPase contains subunits *ac*_8_*c’ c”def*. However, the Vo domain of bovine V-ATPase possesses subunits *ac*_9_*c”de*Ac45, as previously reported^17,27^. Three extra TMs (transmembrane helices) in the center of btVo domain have been observed in the cryo-EM map (Fig. 1c). In order to unambiguously identify these TMs, we combined the results of mass spectrometry, previously reported scVo domain at atomic resolution and of our density map. This analysis revealed that one of these helices is TM1 of subunit *c”*, while TMs 2–5 of subunit *c”* form the c-ring with the nine copies of subunit *c* (designated as *c*_*1*_-*c*_*9*_ counterclockwise from cytosolic view) (Fig. 3a). The second helix in the center of the c-ring belongs to Ac45, which is essential for the activity of V-ATPase^28^; remarkably, the last TM is part of the (pro)renin receptor (PRR) (Figs. 3a, b and 4a), a 39-kD type-I membrane protein, which is involved in various physiological processes like the cell cycle, water homeostasis, and blood pressure regulation^29^. Knocking out the PRR leads to a dysfunctional V-ATPase, interfering with vesicular acidification in organs^30,31^.

In addition to subunit *e2*, two α-helices associated with subunit *a1* have been observed at the edge of the c-ring (Figs. 1c and 3a). Because two analogous α-helices have been identified as subunit *f* in the scVo structure, we followed this nomenclature and named these helices as subunit *f*. To date, the function of subunit *f* is unknown, and knockout of this subunit does not affect the function of V-ATPase in yeast. Mass spectrometry results and our cryo-EM data of the btV-ATPase indicate that subunit *f* may be the RNAseK that was shown to associate with V-ATPase^32^. The specific function of RNAseK in V-ATPase still requires further characterization.

### Interaction details of Vo domain

The N-terminus of Ac45 contains two luminal domains (Fig. 4b). A previous study showed that furin protease can cleave the linker between the two domains^33^. Consistent with this observation, only the second luminal domain and TM domain (residues 251–455) are present in our cryo-EM map. Glycosylation sites in this domain facilitated unambiguous residue assignment (Supplementary Fig. 6c). The second luminal domain of Ac45 was also found in a low-resolution cryo-EM map of bovine V-ATPase, supporting our observation^34^. Similarly, the luminal domain of PRR is not observed in the map, since it has been cleaved by furin protease^35^. The residues 293–313 of renin receptor binds to the luminal domain of Ac45 that contains 14 β-strands and a disulfide bridge between Cys369 and Cys416 (Fig. 4b, c). The structural analysis shows that PRR engages in several hydrophobic interactions with the TM of Ac45 and TM1 of *c”* (Fig. 4c). Besides the hydrophobic contacts between PRR, Ac45, and subunit *c”*, Lys299 of PRR interacts with Gln270 of Ac45 and Glu43 of subunit *c”* have hydrophilic interactions with Gln358 of Ac45 to further stabilize the core of the c-ring (Fig. 4c).

Many phospholipid molecules were observed in the Vo domain, particularly in the lumenal leaflet, indicating that they may play a role in stabilizing the core of the Vo domain (Fig. 3b; Supplementary Fig. 6c). From the density, we can distinguish that these densities belong to the phospholipids, including phosphatidylcholine (PC), phosphatidylethanolamine (PE), phosphatidylserine (PS). The previous studies showed that purified endogenous btV-ATPase could be associated with a mixture for these lipids^36^. Reconstitution of the V-ATPase complex with proton-pumping activity requires the liposomes containing a mixture of PC, PE, PS, and cholesterol^36^. Since we could not identify these phospholipids in each map, we tentatively docked PC as a lipid representative into our cryo-EM map. These lipids may provide a hydrophobic environment to maintain the conformation of the c-ring core (Fig. 3). Interestingly, a sterol-like density was observed at Trp330 of PRR in the cryo-EM map (Fig. 4d), suggesting that a lipid molecule may stabilize the interaction of PRR with subunit *c*_*4*_ in Vo.

### Proton translocation by Vo

**Fig. 5:**
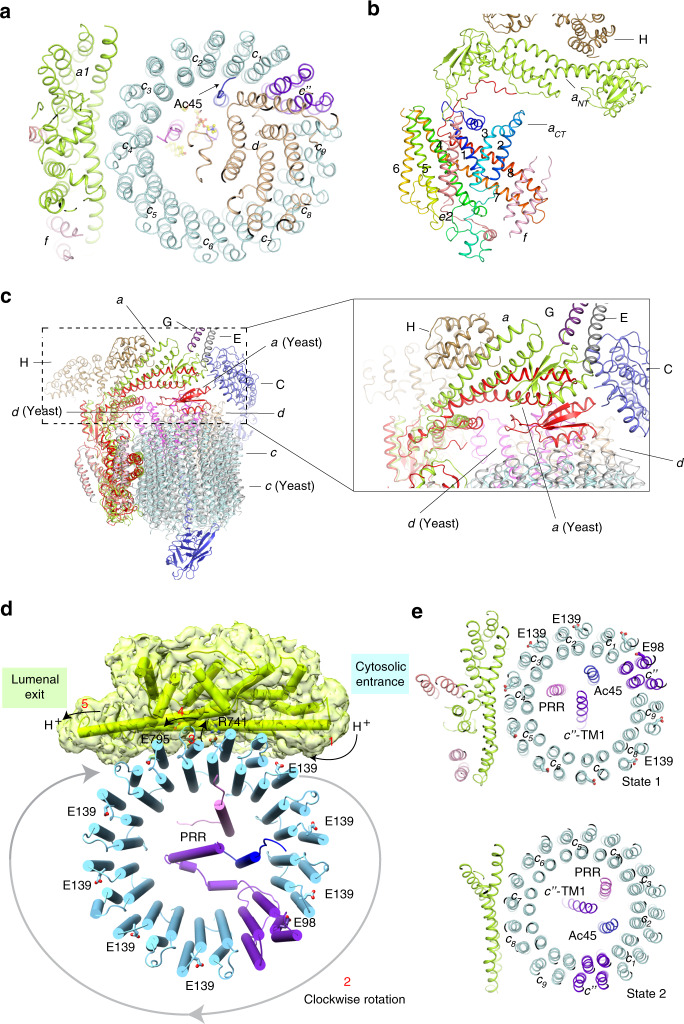
Proton translocation and the rotation of Vo domain. **a** Overall structure showing the connection between subunit d and c-ring. **b** The structural elements of subunit a1 with subunit H, e2, and f. The transmembrane helices of a1 have been labeled. **c** Structural comparison with auto-inhibitory yeast Vo domain (pdb: 6O7T). **d** The putative proton-translocation mechanism. The residues for the proton transfer are shown in sticks. The cryo-EM map of subunit a is shown. **e** The cytosolic view of Vo showing the rotation of c-ring in two states. The Glu residues for protonation in state 1 are shown in stick.

In the state 1, one pair of subunit EG interacts with subunit *a*, while the other pair engages with subunit C, which binds to subunit *d*; moreover, subunit *d* binds to subunits DF to connect the V1 and Vo domains (Fig. 1a). The interaction between subunits *d* and C will be released during the rotation (Fig. 1e). The C-terminal TM of Ac45 interacts with subunit *d* and subunit *c*_*1*_ (Fig. 5a). The subunit *d* also makes several contacts with *c”, c*_*1*_*, c*_*7*_*, c*_*8*_, and *c*_*9*_ (Fig. 5a). These interactions are retained in the two states, suggesting that the fixed conformations of the peripheral subunits can facilitate the rotation of subunits DF*d* and of the c-ring during ATP hydrolysis and proton translocation. These observations imply that the subunit *d* is the key component to connect the c-ring with subunits DF of the V1 domain.

The subunit *a* contains two domains: an N-terminal cytoplasmic region (*a*_*NT*_) and eight C-terminal TMs (*a*_*CT*_) (Fig. 5b). Comparing the structure to yeast Vo, the yeast Vo domain is in an autoinhibit state after dissociation with V1 with subunit *a* N-terminal domain (residues186–258) bound to subunits *c* and *d*, the conformational change on the N-terminus of subunit *a* in non-inhibitory state induces it interacting with subunits H, C, and two peripheral stalks which facilitate the V1 and Vo assembly and ATP hydrolysis coupled proton translocation^37,38^ (Fig. 5c).

There are two aqueous half-channels in subunit *a* near the interface to the c-ring providing access for protons from the cytoplasm to lumen or extracellular space (Fig. 5d). We hypothesize that a proton accesses the lipid bilayer through the cytoplasmic half-channel to neutralize the negative charge of a conserved residue Glu139 of subunit *c* that would directly transfer the proton to Arg741 of subunit *a;* then, the protonated Arg741 would deliver the proton into the luminal/extracellular half-channel^18,20,39–41^. Proton release may be facilitated by the residue Glu795 of subunit *a*, which is conserved in eukaryotic V-ATPases (Fig. 5d).

The structures of the A_3_B_3_ hexamer in the two states showed that ATP hydrolysis triggers a conformational change in the subunit DF, which further induces a shift of subunit *d*. The c-ring is subsequently rotated and results in a continuous proton translocation across the membrane. Notably, Glu98 of the subunit *c”* disrupts the pattern that is created by the key Glu residues of the nine *c* subunits in the c-ring, causing an asymmetric distribution of these Glu residues (Fig. 5e). The structural analysis shows that the TM1 of subunit *c”*, Ac45, and PRR do not change their relative positions in these two states. However, they have rotated ~120^o^ with the c-ring (Fig. 5e), suggesting that the entire c-ring including subunits *c, c”*, Ac45, and PRR function as a rigid domain in proton translocation.

## Discussion

We compared the structures of state 1 and state 2, with the previously reported model of V/A-ATPase^17^ to elucidate the rotational mechanism. We found that the switch of AB heterodimers (Fig. 2b) provides the energy necessary to trigger a rotation of subunits DF resulting in the spinning of subunit *d* coupled with the entire c-ring region, and each state will rotate ~120° (Fig. 5e). Remarkably, the complex interaction network between subunit *d*, TM1 of subunit *c”*, Ac45, PRR, and lipids in our cryo-EM data, provides multiple fulcrums to facilitate the rotation of the entire c-ring region between different states.

Three different rotational states of yeast V-ATPase^16^ and ttV/A-ATPase^17^ had been found in the cryo-EM maps; in contrast, our cryo-EM maps reveal only two rotational states of btV-ATPase (Figs. 1a, d and 5e). The previous structural studies revealed that the endogenous ATPase exhibits the unequal population for three different classes, such as the yeast V-ATPase^16^ with state 1 (47%, 6.9 Å), state 2 (36%, 7.6 Å), state 3 (17%, 8.3 Å), and the ttV/A-ATPase^42^ with state 1 (66%, 5.0 Å), state 2 (16%, 6.7 Å), state 3 (7%, 7.5 Å), and 11% bad class. These findings imply that state 3 shows the least population and the worst resolution after 3D refinement. We speculate that the third state, where the subunit *c”* faces subunit *a*, may not be stable in our bovine tissue preparation or exists in very few populations that could not be captured, as the similar results observed in some studies^42,43^.

The structures of rat V-ATPase with SidK, a bacterial inhibitor of V-ATPase, has been reported recently^20^. This remarkable work showed that Ac45, PRR, and RNAseK are essential components of Vo, consistent with our analysis. In the rat V-ATPase structures, three conformationally different AB heterodimers have been reported: one is bound to ADP and the other two represent nucleotide-free states. When comparing our structure with the rat V-ATPase structure, we find that their conformations of AB_open_ and AB_occluded_ share similar conformations with our AB heterodimers (Supplementary Fig. 9a, b); however, the C-terminal α-helical domain of subunit A in the AB_closed_ state of SidK bound V-ATPase has a ~3 Å shift to make the AB_closed_ state more compact than the active form (Supplementary Fig. 9c). The structural comparison reveals that the subunits EG have a ~4 Å shift between the two structures; this difference may be caused by the presence of subunit H that has contact with the subunits EG directly (Supplementary Fig. 9d). The V1 and Vo structures of rat V-ATPase adopt a similar conformation to those in bovine V-ATPase structures state 1 and state 2, respectively. Due to the limitation of the resolution of the rat V-ATPase structure, the luminal domain of Ac45 could not be modeled; in contrast, we built the entire second luminal domain of Ac45 showing the interaction details of the Ac45 and PRR (Fig. 4c; Supplementary Fig. 9e). In summary, we report the mammalian V-ATPase structure including all the essential subunits, revealing a more complete map of this important rotary machine.

## Methods

### Gain clathrin-coated vesicles

About three defatted bovine brains were rinsed and blended with 0.1 M Na-MES, pH 6.5, 1 mM EGTA, 0.5 mM MgCl_2_, and 3 mM NaN_3_ (buffer A). Usually, one kilogram of the cleaned tissue is homogenized in 900 ml of buffer A. The homogenate is centrifuged in a GSA (Sorvall) rotor for 50 min at 20,000 *g*, and the supernatant is centrifuged at 140,000 *g* for 1 h to sediment the membrane vesicles. The pellet containing enriched coated vesicles is resuspended in buffer A at a protein concentration of 15 mg/ml, frozen in liquid nitrogen, and stored at −80 °C.

### Purification of V-ATPase

The V-ATPase was solubilized and purified using an established protocol^21^. Briefly, the membrane vesicles incubated with 0.75 M Tris-HCl pH 8.0 on ice for 30 min to strip clathrin. After centrifugation at 150,000 *g* for 35 min, the pellet was resuspended in 0.5% Na-cholate and incubated at 0 °C for 30 min. After centrifugation at 150,000 *g* for 35 min, the pellet was resuspended in 0.75% C_12_E_9_, 10 mM Tris-MES pH 6.75, incubated on ice for 60 min and centrifuged at 150,000 *g* for 90 min.

The supernatant was applied to a hydroxylapatite column which had been equilibrated with 0.1% C_12_E_9_, 10% glycerol, 0.5 mM DTT, 10 mM Tris-MES pH 7.0 (buffer B). The column was washed with 2CV of buffer B and eluted with 0–0.3 M Na-phosphate in buffer B. Saturated ammonium sulfate solution was added dropwise to the active hydroxylapatite fractions (selected by either SDS-PAGE or ATPase assay), to a final concentration of 1.65 M. The mixture was centrifuged at 100,000 *g* for 30 min, the pellet was harvested and dissolved in buffer B without glycerol.

Then the sample was loaded on to a glycerol gradient (12 ml, 10–30%) prepared in buffer B. The gradient was centrifuged at 170,000 *g* for 20 h, and fractions were collected. The fractions containing V-ATPase were concentrated and further purified by gel filtration using a Superose 6 10/300 column (GE Healthcare) pre-equilibrated with buffer C (20 mM HEPES pH 7.5, 150 mM NaCl, 0.1% CHAPS, 0.004% glycodiosgenin (Anatrace)). Mass spectrometry confirmed the identity of the subunits of V-ATPase (Supplementary Table 1). The peak fractions were collected and concentrated to 2.5–3.5 mg/ml for cryo-EM grid preparation.

### V-ATPase activity assay

ATPase activity was measured as the liberation of ^32^Pi from [γ−^32^P]ATP^21^. The assay was carried out in a total volume of 200 μl under the following conditions: 4 μl phosphatidylserine (26 μM concentration), 5 μl V-ATPase in buffer C (at 10 nM concentration), and 189 μl ATPase assay solution A (30 mM KCl, 50 mM Tris-MES, pH 7.0, 3 mM MgCl_2_, and 3 mM [γ−^32^P]ATP (400 cpm/nmol)) and 2 μl of ethanol or ethanol dissolved bafilomycin A1 (Sigma-Aldrich, 1 μM concentration), The V-ATPase was first incubated with phosphatidylserine for 2 min, and then the reaction was started by addition ATPase assay solution A and ethanol/bafilomycin A1, and continued for 10 min at 37 °C. The ATP hydrolysis reaction was terminated by adding 1.0 ml of 1.25 N perchloric acid, and the released ^32^Pi was extracted and counted in a Beckman scintillation counter^44^. The results were expressed as specific activity (μmol of Pi/min per mg of protein), The experiment has been repeated twice, and the results were performed using GraphPad Prism8.

### EM sample preparation and imaging for 200 kV Cryo-TEM

Freshly purified 2.5–3.5 mg/ml V-ATPase in buffer C was applied to Quantifoil R1.2/1.3 300 mesh Au holey carbon grids (Quantifoil). The grids were then blotted and plunged into liquid ethane for flash freezing using a Vitrobot Mark IV (FEI). The grids were imaged in a 200 keV Talos Arctica (FEI) with a Gatan K3 Summit direct electron detector (Gatan) in super-resolution mode using the data-collection software Serial EM. Data were collected at 0.735 Å/pixel with a counted rate of 42 electrons per physical pixel per second. Images were recorded for 4 s exposures in 50 subframes with a total dose of 80 electrons per Å^2^ and a defocus range of −1.0 to −2.0 μm.

### Imaging processing for 200 kV Cryo-TEM

Dark-subtracted images were first normalized by gain reference and then binned twofold that resulted in a pixel size of 1.47 Å. Motion correction and gain reference were performed using the program MotionCor2^45^. The contrast transfer function (CTF) was estimated using CTFFIND4^46^. To generate V-ATPase templates for automatic picking, around 2000 particles were manually picked and classified by 2D classification in RELION-3^47^. After auto-picking, the low-quality images and false-positive particles were removed manually. The remaining 420,602 particles were extracted for subsequent 2D. A low-resolution cryo-EM map of V-ATPase, which was generated from 3200 particles by RELION-3, was used as the initial model for 3D classification. The best two classes, containing 75,386 particles, provided a 8.53 Å map after 3D auto-refinement with a mask and postprocess in RELION-3, then CTF refinement and Bayesian polishing of particles were performed using RELION-3 for once followed by 3D refinement using a soft mask. Next, the second 3D classification was performed, two rotational states were separated by the obvious position of subunits D and F. One class of state 1, including 61,520 particles, provided a 7.61 Å map after 3D auto-refinement with a mask and postprocess in RELION-3. After the second CTF refinement with beam tilt correction, the resulting particles of state 1 were used for the final 3D refinement with a soft mask and solvent-flattened FSCs yielded a reconstruction at 7.22 Å revealing clear secondary structural elements. The resolution was estimated using “post-processing” with the FSC criteria of 0.143.

### EM sample preparation and imaging for 300 kV Cryo-TEM

Freshly purified 2.5–3.5 mg/ml V-ATPase in buffer C was applied to Quantifoil R1.2/1.3 300 or 400 mesh Au holey carbon grids (Quantifoil). The grids were then blotted and plunged into liquid ethane for flash freezing using a Vitrobot Mark IV (FEI). The grids were imaged in a 300 keV Titan Krios (FEI) with a Gatan K3 Summit direct electron detector (Gatan) in super-resolution mode using the data-collection software Serial EM.

Dark-subtracted images collected at super-resolution mode were first normalized by gain reference and binned twofold, which resulted in a pixel size of 0.833 Å with a counted rate of 23 electrons per physical pixel per second^48^. Images were recorded for 1.8-s exposures in 60 subframes with a total dose of 60 electrons per Å^2^ and a defocus range of −1.0 to −2.0 μm.

### Imaging processing and 3D reconstruction for 300 kV Cryo-TEM

The images were collected in two sessions (images from 400 mesh, images from 300 mesh Au holey carbon grids). The images used for the second session were divided into two parts for data processing. Dark-subtracted images were first normalized by gain reference and then binned twofold that resulted in a pixel size of 0.833 Å. Motion correction and gain reference was performed using the program MotionCor2^45^. The contrast transfer function (CTF) was estimated using CTFFIND4^46^. To generate V-ATPase templates for automatic picking, about 2000 particles were manually picked and classified by 2D classification in RELION-3^47^.

After auto-picking, low-quality images and false-positive particles were removed manually. About 112 K/174 K/210 K particles of V-ATPase were extracted. We used the cryo-EM structure of V-ATPase, which was determined by us with the data collected from a 200 keV Arctica (FEI) low-pass filtered to 40 Å as the initial model and applied a mask for 3D classification. The best class, containing 28,125/45,034/56,149 particles, provided a 4.29 Å /5.00 Å/4.88 Å map after 3D auto-refinement with a mask and postprocess in RELION-3.

For the particles in each dataset, CTF refinement and Bayesian polishing of particles were performed using RELION-3 for once followed by 3D refinement using a soft mask. Then, we combined the particles from CTF refinement of dataset 1, the particles from CTF refinement of dataset 2, and particles from Bayesian polishing of dataset 3 with a total of 129,308 particles and performed a 3D classification with six classes. Two rotational states were separated by the obvious position of subunits D and F. Two classes of state 1 including 84,345 particles refined using a soft mask and solvent-flattened Fourier shell correlations (FSCs) yielded a reconstruction at 3.45 Å. Applying a soft mask in RELION-3 post processing yielded a final cryo-EM map of 3.37 Å. One class of state 2 including 41,821 particles refined using a soft mask and solvent-flattened Fourier shell correlations (FSCs) yielded a reconstruction at 3.86 Å. Applying a soft mask in RELION-3 post processing yielded a final cryo-EM map of 3.79 Å. Resolution was estimated using the Fourier shell correlation (FSC) 0.143 criterion.

For rotational state 1, after 3D refinement, applying a full mask in RELION-3 post processing yielded a 3.37 Å overall resolution based on the Fourier shell correlation (FSC) 0.143 criterion. But due to the flexibility of V1 and Vo, Vo was not well resolved. Several focused refinements with different masks were attempted to get a high-quality local map. These focused refinements included subunits A_3_B_3_DE_3_FG_3_, subunits A_3_B_3_CDE_3_FG_3_H, subunits *ad*CFH, subunits CFHVo provided some good local maps for this state, especially for the Vo part (Supplementary Fig. 5). After post processing, these refinements gave a resolution of 3.22 Å, 3.26 Å, 3.89 Å, and 3.54 Å, respectively. For rotational state 2, after 3D refinement, applying a full mask in RELION-3 post processing yielded a 3.79 Å overall resolution based on the Fourier shell correlation (FSC) 0.143 criterion. The same strategy had been applied to improve the local maps of state 2. These focused refinements included subunits A_3_B_3_DE_3_FG_3_, subunits A_3_B_3_CDE_3_FG_3_H, subunits CFHVo, subunits *c*_9_*c”def*Ac45 PRR, provided good local maps for this state. After post processing, these refinements gave a resolution of 3.60 Å, 3.79 Å, 4.47 Å, and 3.73 Å, respectively. All these focused maps of rotational state 1 and state 2 can be used to generate a composite map for refinement.

### Model construction

To obtain better side-chain densities for model building, we sharpened the map of rotational state 1 of V-ATPase using post processing in RELION-3 with a B-factor value of −79.3 Å^2^. Based on the previously reported structures including subunit AB (PDB code 5ZE9), subunit C (PDB code 1U7L), subunit D (PDB code 4RND), subunits EG (PDB code 4EFA), subunit F (PDB code 4IX9), subunit H (PDB code 1HO8), and subunits *a, c, c”, d, e* (PDB code 6O7U), the homology models for subunits A-H of V1 and subunits *a, c, c”, d, e*, of Vo were generated by MODELLER^22^, Ac45 and subunit *f* were predicted by Phyre2^49^. Each subunit was docked into the cryo-EM map, respectively, using Chimera^50^ to generate the initial model, and then manually adjusted using COOT^51^. The transmembrane domain of PRR was built de novo. The glycosylation sites of Ac45 luminal domain were assigned to facilitate the register of Ac45. The residues of bovine V-ATPase that were not resolved or built are shown in Supplementary Table 1. For rotational state 2, we sharpened the map of the complex using post processing in RELION-3 with a B-factor value of −85.8 Å^2^. Atomic models of each subunit were based on the structure of rotational state 1. Each subunit was docked into the cryo-EM map, respectively, using Chimera^50^ to generate the initial model, and then manually adjusted by COOT^51^. Besides the unmodelled residues in state 1, the densities of subunit *a* (residues 4–8, 167–169, 382–529, 564–658, 830–834), subunits *e* and *f* were not resolved nor built. Side chains were removed to generate a model for subunit C (residues 42–310, 325–343), subunit H and subunit *a* (residues 530–563) due to the limited local resolution.

### Model refinement and validation

For state 1, to generate a composite map for refinement, several focused maps that have been described above were combined and aligned using phenix.combine_focused _maps which would coalescence the best parts of several maps together. The model was refined in real space using PHENIX^52^ with secondary-structure restraints and stereochemical restraints^53^. The same strategy has also been used for state 2. For cross-validations, the final model was refined against one of the half maps generated by 3D auto-refine, and the model vs. map FSC curves were generated in the comprehensive validation module in PHENIX. MolProbity^54^ and PHENIX were used to validate the final model. Local resolutions were estimated using RELION-3. Structure Figures were generated using PyMOL and Chimera^50^.

### Reporting summary

Further information on research design is available in the Nature Research Reporting Summary linked to this article.

## Supplementary information

Supplementary Information

Reporting Summary

## Data Availability

Data supporting the findings of this paper are available from the corresponding authors upon reasonable request. A reporting summary for this Article is available as a Supplementary Information file. The 3D cryo-EM density maps of rotational state 1 and state 2 of V-ATPase have been deposited in the Electron Microscopy Data Bank under the accession numbers EMD-22121 and EMD-22122. Atomic coordinates for the atomic model of rotational state 1 and state 2 of V-ATPase have been deposited in the Protein Data Bank under the accession numbers PDB 6XBW and PDB 6XBY. Source data are provided with this paper.

## Source data

Source Data
