# Supplementary material for: Cryo-EM structures of intact V-ATPase from bovine brain
Source: Nat Commun. 2020 Aug 6;11:3921. doi: 10.1038/s41467-020-17762-9 (PMC7414150; doi:10.1038/s41467-020-17762-9)
Supplement: Supplementary file 1 — Supplementary Information [file 41467_2020_17762_MOESM1_ESM.pdf]

Cryo-EM structures of intact V-ATPase from bovine brain

Rong Wang, Tao Long, Abdirahman Hassan, Jin Wang, Yingyuan Sun, Xiao-Song Xie and Xiaochun Li

Correspondence and requests for materials should be addressed to X.X. (email: xiao-song.xie@utsouthwestern.edu) or to X.L. (email: xiaochun.li@utsouthwestern.edu)

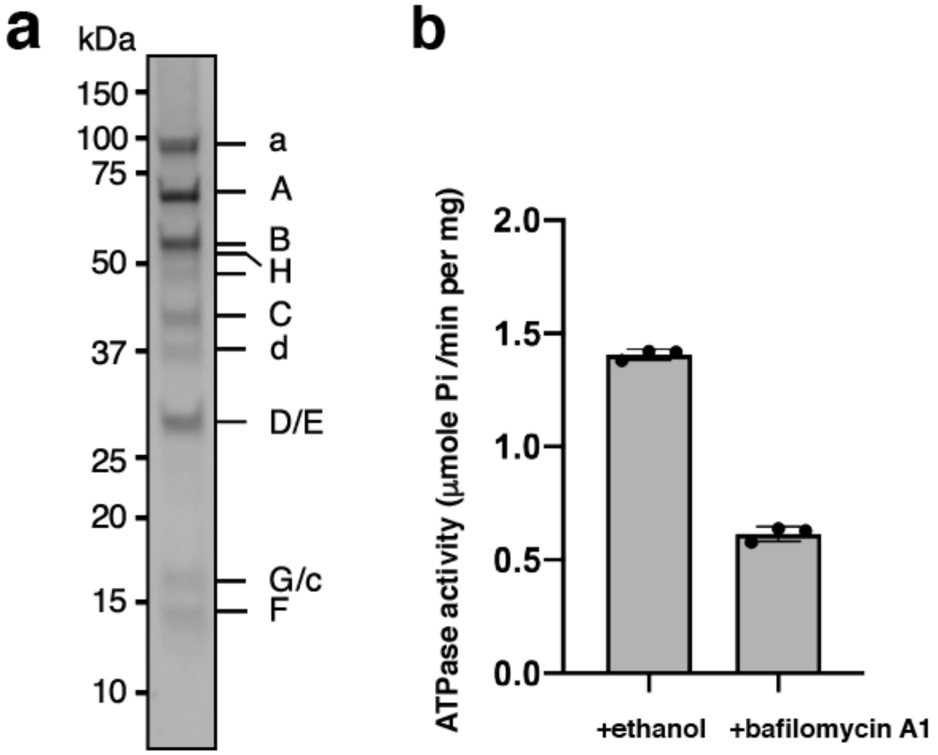

Supplementary Fig. 1 V-ATPase activity assay.

a. SDS-PAGE for V-ATPase from endogenous bovine brain. **b.** The purified bovine V-ATPase in 20 mM Hepes pH 7.5, 150 mM NaCl, 0.1% CHAPS, 0.004% glyco-diosgenin was used for V-ATPase activity assay, and the activity was measured as liberation of ^{32}Pi from [γ-^{32}P] ATP as described in methods. The addition of bafilomycin A1 at 1 μM decreased the V-ATPase activity comparing with WT. Each bar represents the mean of triplicate determination of the experiment repeated at least twice. Data are mean ± s.d. Source data are provided as a source data file.

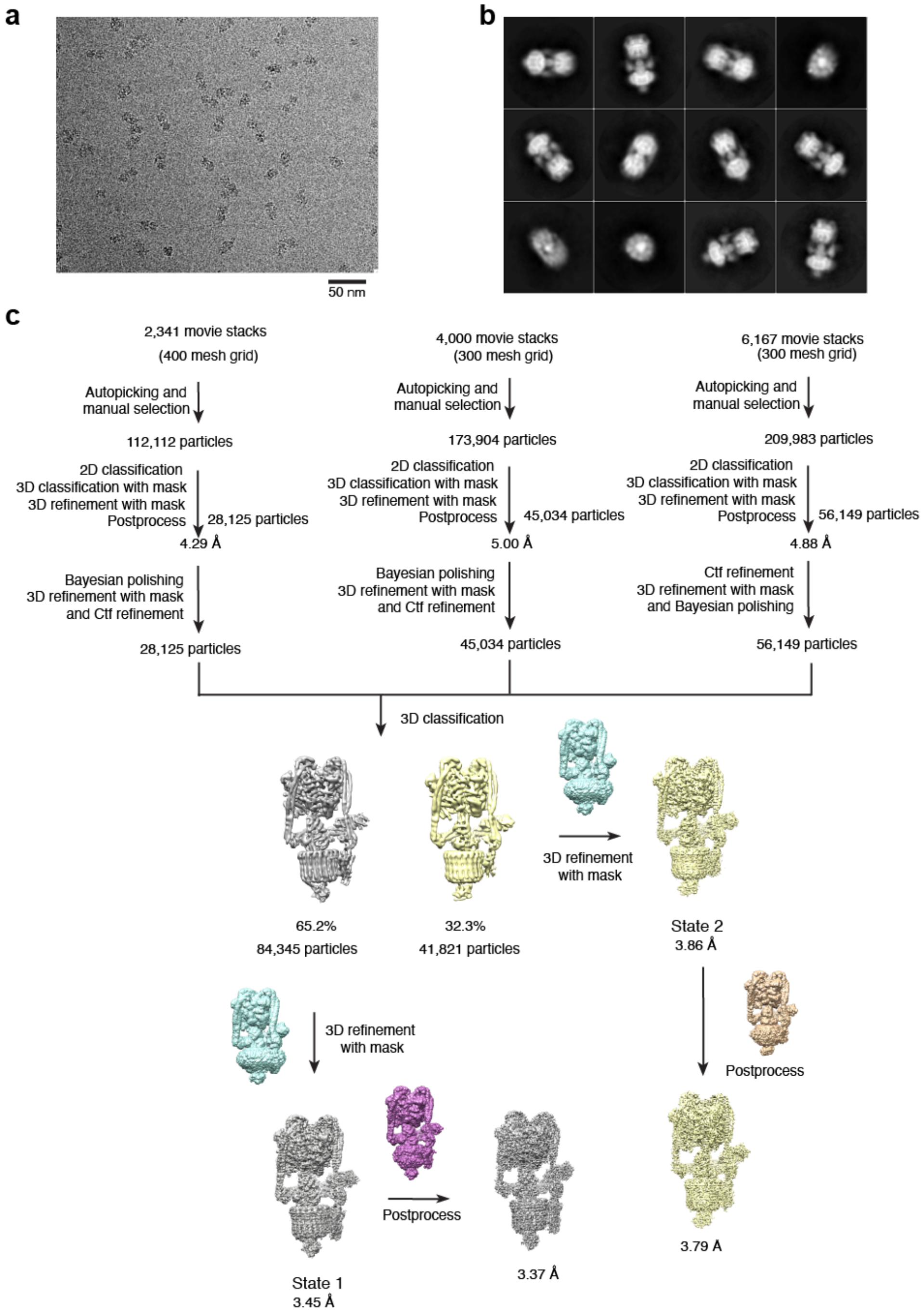

Supplementary Fig. 2 Data processing.

a. A representative electron micrograph at -2.0 μm defocus. **b.** The cryo-EM 2D classification from RELION is shown. **c.** The data processing work-flow.

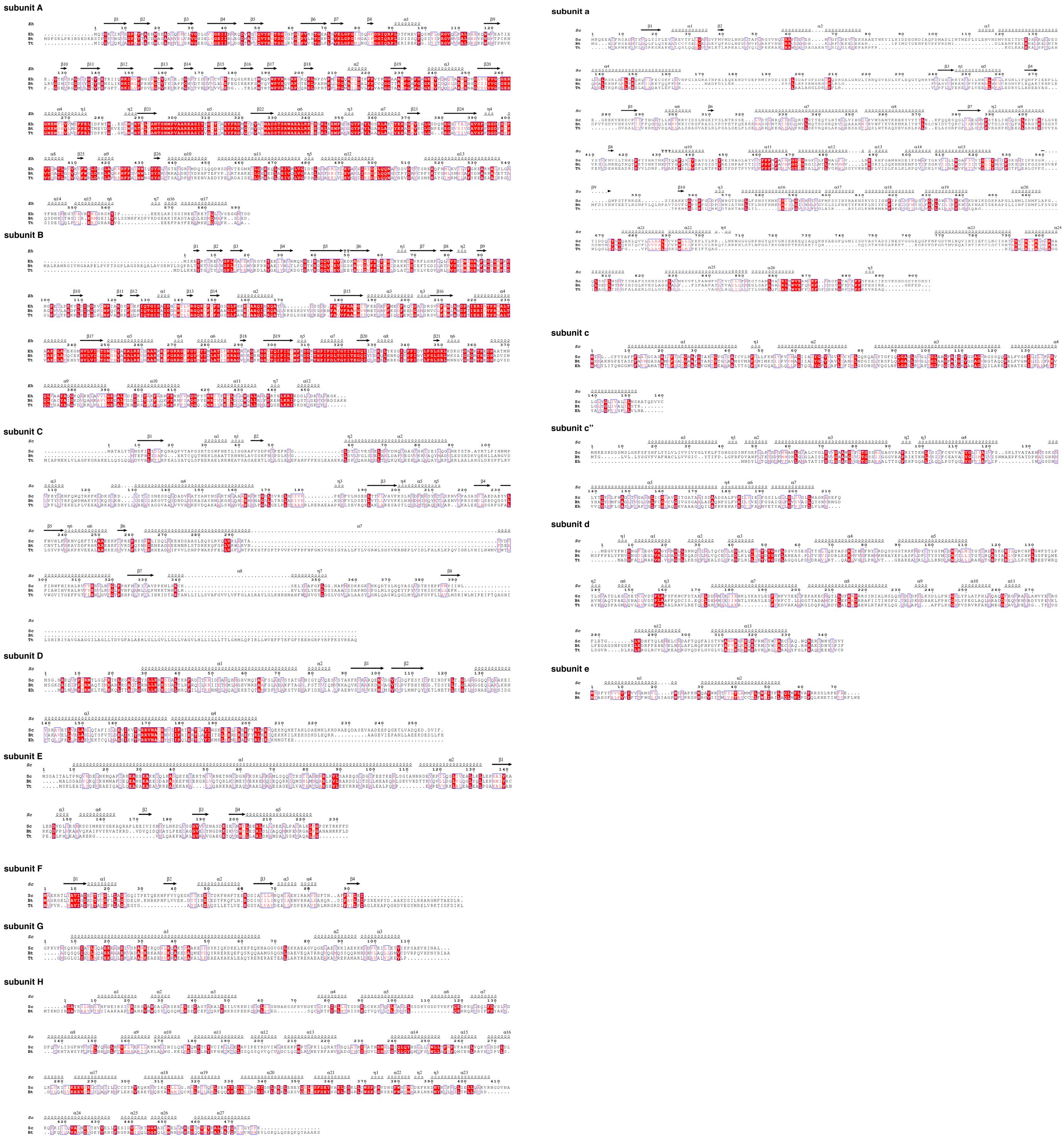

Supplementary Fig. 3 Sequence alignment of models of individual subunits of bovine V-ATPase that generated from the MODELLER software by Espript 3.0.

The first sequence is the model we refer to for each subunit modeling as described in method, the second sequence is our bovine sequence, and the third sequence is an alternative model from other species with lower rank. Eh, Bt, Tt, Sc represent *Enterococcus hirae*, *Bos taurus*, *Thermus thermophilus*, *Saccharomyces cerevisiae*, respectively. For subunit A and B, the Eh model is from PDB-5ZE9, the Tt model is from PDB-3GQB; For subunit C, the Sc model is from PDB-1U7L, the Tt model is from PDB-6R0W; For subunit D, the Sc model is from PDB-4RND, the Eh model is from PDB-3AON; For subunit E and G, the Sc model is from PDB-4EFA, the Tt model is from PDB-3V6I; For subunit F, the Sc model is from PDB-4IX9, the Tt model is from PDB-2D00; For subunit H, the Sc model is from PDB-1HO8; For subunit *a*, the Sc model is from PDB-6O7U, the Tt model is from PDB-6R0W; For subunit *c* and *c''*, the Sc model is from PDB-6O7U, the Eh model is from PDB-2BL2; For subunit *d*, the Sc model is from PDB-6O7U, the Tt model is from PDB-1V9M; For subunit *e*, the Sc model is from PDB-6O7U. Strictly conserved residues are highlighted in shaded red boxes and conserved residues in open red boxes. The secondary structures of the model are placed on the top of the alignments.

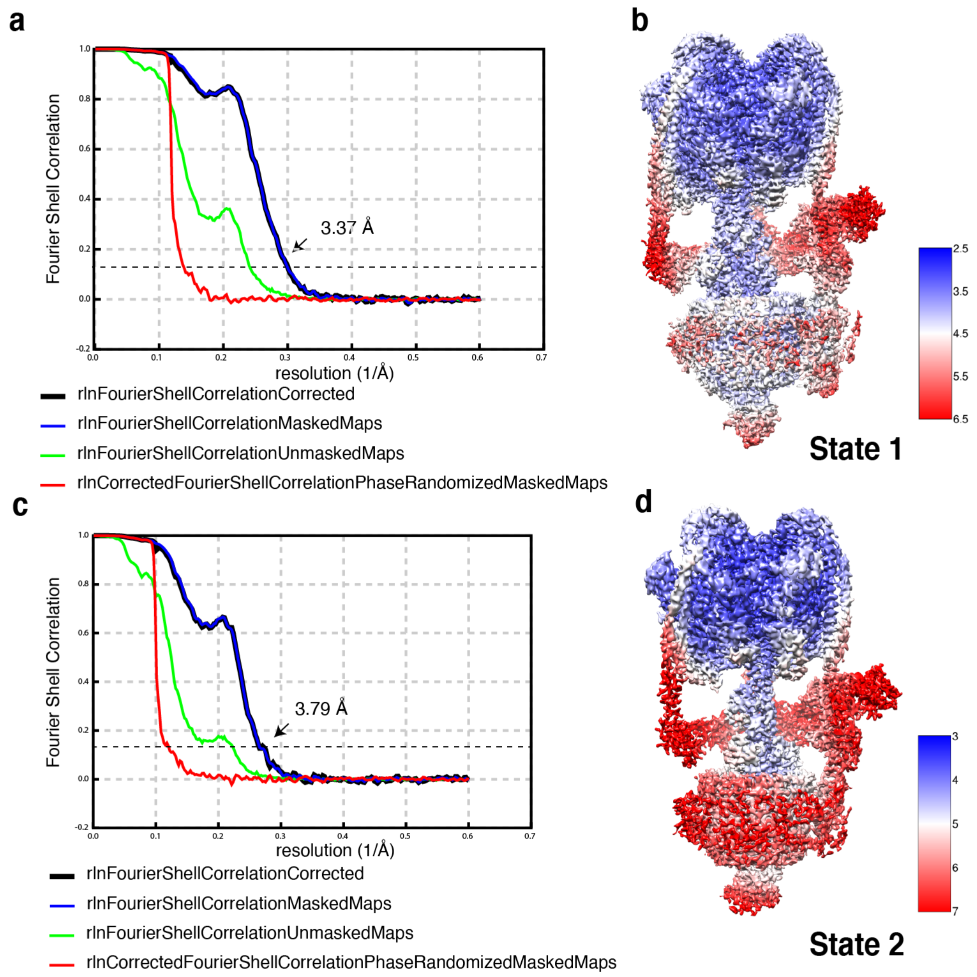

Supplementary Fig. 4 FSC curve and local resolution estimation of the intact V-ATPase in the two states.

a. Fourier shell correlation (FSC) curve as a function of resolution of state 1 using RELION-3 output. **b.** Density maps of state 1 structure colored by local resolution estimation using RELION-3. **c.** Fourier shell correlation (FSC) curve as a function of resolution of the state 2 using RELION-3 output. **d.** Density maps of state 2 structure colored by local resolution estimation using RELION-3.

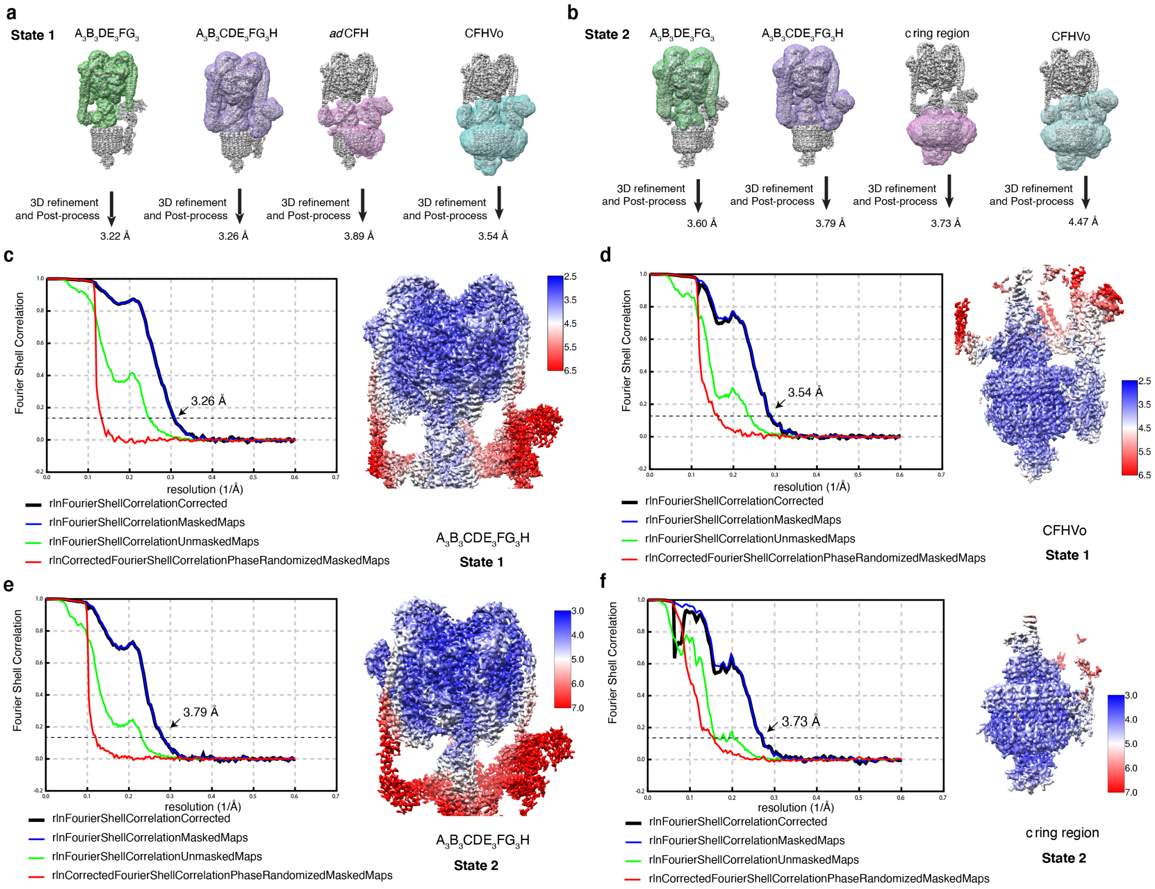

Supplementary Fig. 5 The focused maps and their FSC curves, local resolution estimation of the focused map of V1 and Vo domains in the two states.

a. and **b**. The masked regions for focused refinement. **c.** and **d**. Fourier shell correlation (FSC) curve as a function of resolution of the V1 of the state 1 (**c**) and the Vo of the state 1 (**d**) using RELION-3 output. Density maps of the V1 of the state 1 (**c**) and the Vo of the state 1 (**d**) are colored by local resolution estimation using RELION-3. **e.** and **f**. Fourier shell correlation (FSC) curve as a function of resolution of the V1 of the state 2 (**e**) and the Vo of the state 2 (**f**) using RELION-3 output. Density maps of the V1 of the state 2 (**e**) and the Vo of the state 2 (**f**) are colored by local resolution estimation using RELION-3.

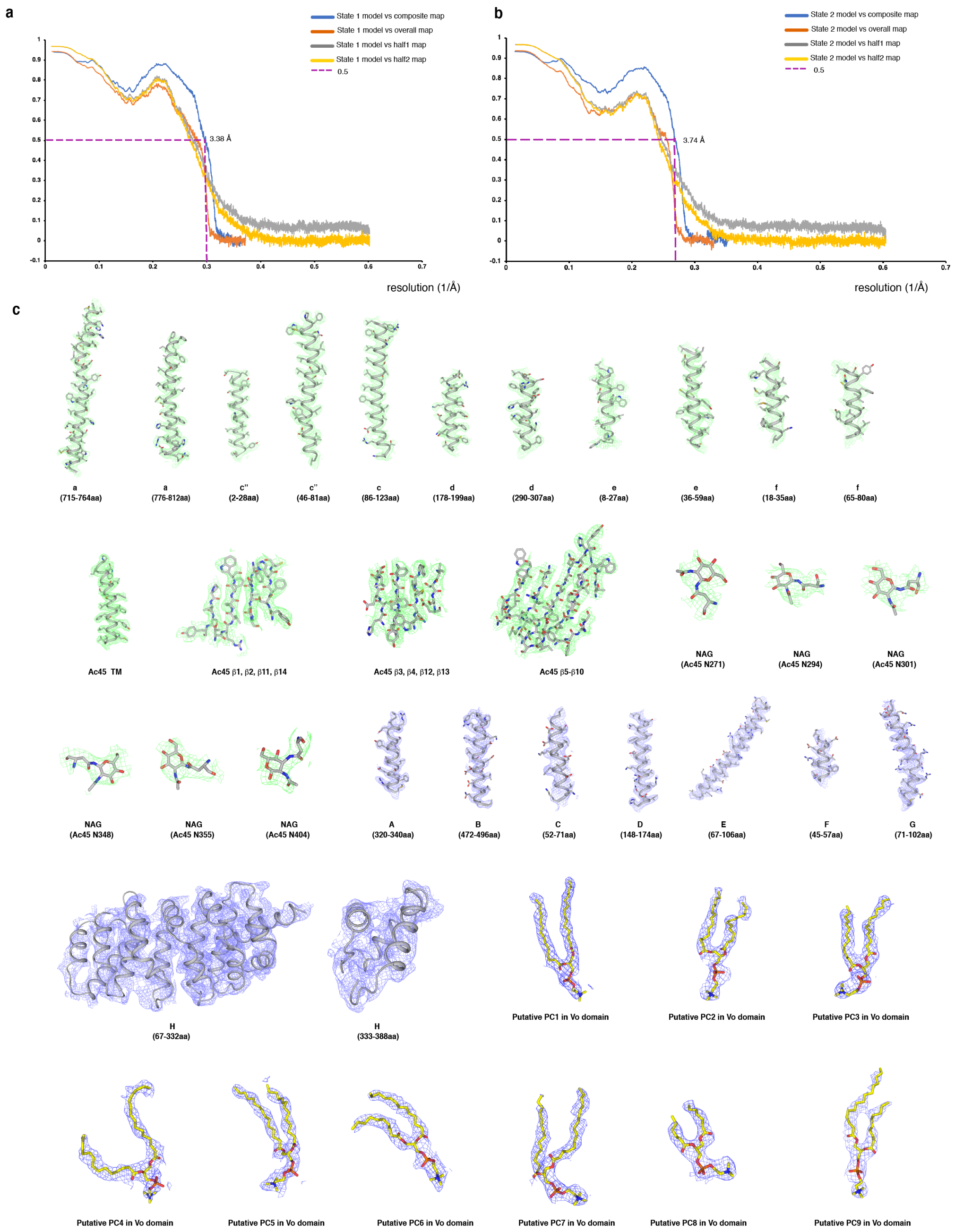

Supplementary Fig. 6 Cryo-EM map of structural elements.

a. and **b.** The FSC curves of state 1 (**a**) and state 2 (**b**) calculated between the refined structure model and the composite map (blue) used for refinement, the overall half map (gray), the other half map (yellow) and the overall full map (orange). **c.** The structural elements of the state 1 with cryo-EM map. The maps are shown at 5σ level.

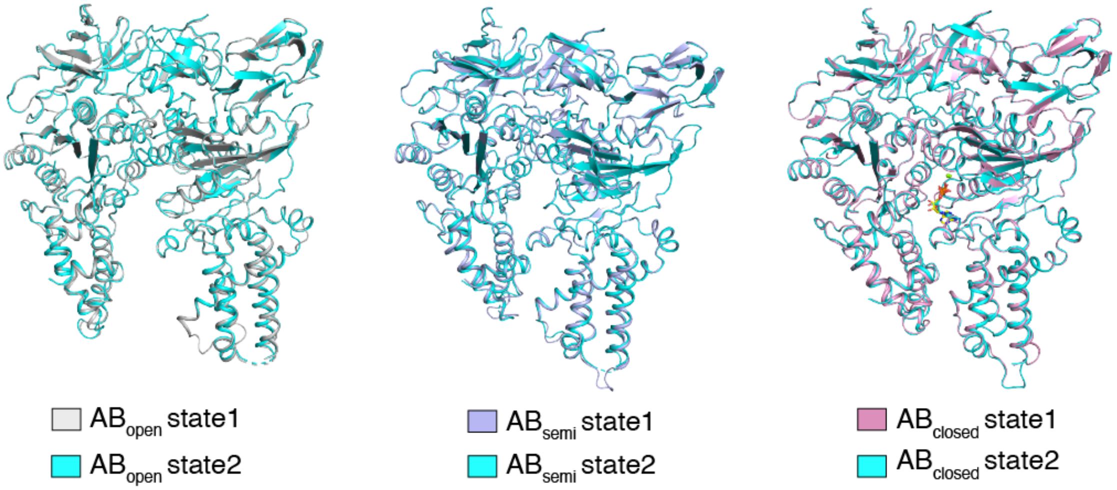

Supplementary Fig. 7 Structural comparison of AB heterodimers in two states.

The AB heterodimers in the state 2 are colored in cyan.

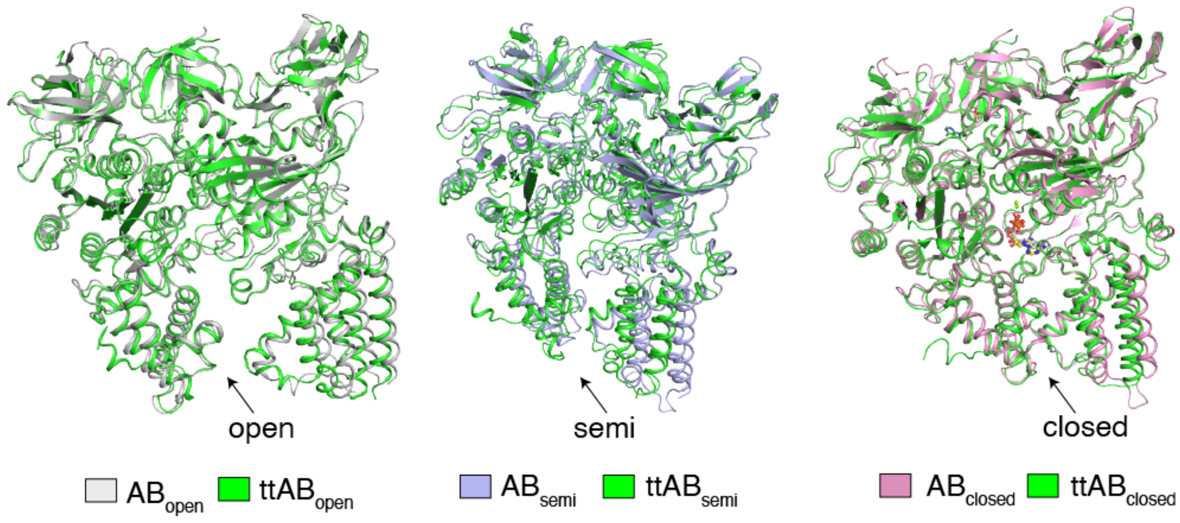

Supplementary Fig. 8 Structural comparison of AB heterodimers with ttV/A-ATPase.

The AB heterodimers in ttV/A-ATPase are colored in green.

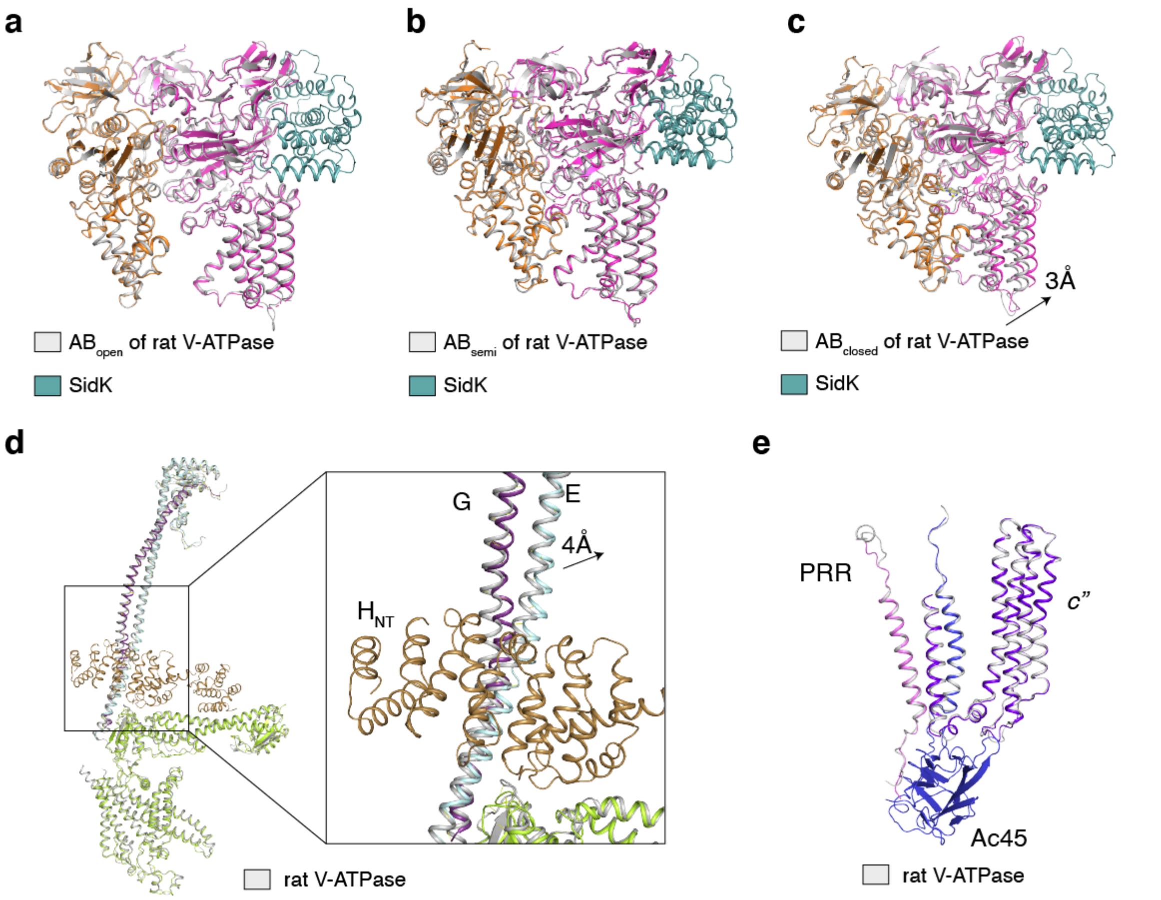

Supplementary Fig. 9 Structural comparison of bovine V-ATPase with rat V-ATPase (pdb code: 6VQ6).

a-c. Structural comparison of AB heterodimers. **d.** The conformations of subunits E, G and H. **e.** The conformations of subunits PRR, *c”* and Ac45. The subunits in rat V-ATPase are colored in gray and SidK is colored in teal.

Supplementary Table 1 Summary of each subunit of state1 model.

Subunit	Chain	Total Residues Built	Remove Side Chains	Unmodelled	Mass Spectrometry Accession	Coverage of Peptides (%)
A	A	16-612		1-15, 558-565, 613-617	P31404	65.48
A	B	16-612		1-15, 251-257, 613-617	P31404	65.48
A	C	16-612		1-15, 613-617	P31404	65.48
B	D	38-506		1-37, 217-224, 507-511	P31408	90.61
B	E	38-506		1-37, 214-224, 507-511	P31408	90.61
B	F	38-506		1-37, 215-224, 507-511	P31408	90.61
C	G	2-379		1, 12-17, 81-85, 345-365, 380-382	P21282	52.36
D	H	6-216		1-5, 217-247	A0A3Q1M4W9	42.91
E	I	6-223	6-53	1-5, 224-226	P11019	71.24
E	J	6-223	6-50	1-5, 224-226	P11019	71.24
E	K	4-223	4-59	1-3, 224-226	P11019	71.24
F	L	5-107		1-4, 108-119	Q28029	53.78
G	M	6-113	6-70	1-5, 114-118	Q0VCV6	51.69
G	N	6-113	6-70	1-5, 114-118	Q0VCV6	51.69
G	O	6-113	6-70	1-5, 114-118	Q0VCV6	51.69
H	P	66-445	72-107, 120-132	1-65, 108-119, 446-476	F1MZL6	55.91
a	a	4-834		1-3, 141-166, 659-713, 835-838	F1MJV0	35.44
c’’	b	2-205		1	ND	
d	d	4-351		1-3	P61420	35.9
e	e	8-80		1-7, 81	ND	
f	f	16-87		1-15, 37-63, 88-98	Q3ZC23	6
c	c	6-155		1-5	P23956	36.13
c	g	6-155		1-5	P23956	36.13
c	k	6-155		1-5	P23956	36.13
c	l	6-155		1-5	P23956	36.13
c	m	6-155		1-5	P23956	36.13
c	n	6-155		1-5	P23956	36.13
c	o	5-155		1-4	P23956	36.13
c	p	5-155		1-4	P23956	36.13
c	q	5-155		1-4	P23956	36.13
PRR	r	293-338		1-292, 339-351	P81134	20.51
Ac45	s	251-455		1-250, 456-468	P40682	13.03

Supplementary Table 2 Cryo-EM data collection, refinement and validation statistics

	State 1 (EMDB-EMD-22121) (PDB-6XBW)	State 2 (EMDB-EMD-22122) (PDB-6XBY)
**Data collection and processing**		
Magnification	60024	
Voltage (kV)	300	
Electron exposure (e–/Å^2)	60	
Defocus range (μm)	-1.0 to -2.0	
Pixel size (Å)	0.833	
Symmetry imposed	C1	
Initial particle images (no.)	495,999	
Final particle images (no.)	84,345	41,821
Overall map resolution (Å)	3.37	3.79
FSC threshold	0.143	0.143
**Refinement**		
Initial model used (PDB code)	5ZE9, 1U7L, 4RND 4EFA, 4IX9, 1HO8 6O7U	State 1
Model resolution (Å)	3.38	3.74
FSC threshold	0.5	0.5
Map sharpening *B* factor (Å^2)	-79.3	-85.8
Model composition		
Non-hydrogen atoms	63090	57518
Protein residues	8186	7765
Ligands	18	15
B factors (Å^2)		
Protein	70.07	74.75
Ligand	51.57	62.56
R.m.s. deviations		
Bond lengths (Å)	0.010	0.006
Bond angles (°)	1.259	1.039
Validation		
MolProbity score	1.68	1.76
Clashscore	8.21	6.94
Poor rotamers (%)	0.60	0.41
Ramachandran plot		
Favored (%)	96.46	94.38
Allowed (%)	3.27	5.46
Disallowed (%)	0.27	0.16
